# Data on selected antimalarial drug resistance markers in Zambia

**DOI:** 10.1016/j.dib.2020.106650

**Published:** 2020-12-13

**Authors:** Lungowe Sitali, Mulenga C. Mwenda, John M. Miller, Daniel J. Bridges, Moonga B. Hawela, Busiku Hamainza, Mutinta Mudenda-Chilufya, Elizabeth Chizema-Kawesha, Rachel Daniels, Thomas P. Eisele, Audun H. Nerland, James Chipeta, Bernt Lindtjorn

**Affiliations:** a Centre for International Health, Faculty of Medicine, University of Bergen, Bergen, Norway; b University of Zambia, School of Health Sciences, Department of Biomedical Sciences, Lusaka, Zambia; c School of Medicine and University Teaching Hospital Malaria Research Unit, University of Zambia, Lusaka, Zambia; d PATH-Malaria Control and Elimination Partnership in Africa, National Malaria Elimination Centre, Ministry of Health, Lusaka, Zambia; e National Malaria Elimination Centre, Ministry of Health, Lusaka, Zambia; f End Malaria Council, African Leaders Malaria Alliance, National Malaria Elimination Centre, Ministry of Health, Lusaka, Zambia; g Broad Institute of MIT and Harvard, Cambridge, MA, USA; h Centre for Applied Malaria Research and Evaluation, Department of Tropical Medicine, Tulane University School of Public Health and Tropical Medicine, New Orleans, Louisiana; i Department of Clinical Science, Faculty of Medicine, University of Bergen, Norway; j University of Zambia School of Medicine, Department of Paediatrics and Child Health, Lusaka, Zambia

**Keywords:** Sulfadoxine-pyrimethamine, Mutations, Zambia, *Plasmodium falciparum*, Malaria

## Abstract

This article describes data on selected resistance markers for antimalarial drugs used in Zambia. Antimalarial drug resistance has hindered the progress in the control and elimination of malaria. Blood samples were collected during a cross-sectional household survey, conducted during the peak malaria transmission, April to May of 2017. Dried blood spots were collected during the survey and transported to a laboratory for analysis. The analysed included polymerase chain reaction (PCR) followed by high resolution melt (HRM) for mutations associated with Sulfadoxine-pyrimethamine resistance in the *Plasmodium falciparum dihydrofolate reductase (Pfdhfr)* and *P. falciparum dihydropteroate synthase (Pfdhps)* genes. Mutations associated with artemether-lumefantrine resistance in *falciparum multi-drug resistance gene 1* (*Pfmdr1*) were also assessed using PCR and HRM analysis, whereas the *P. falciparum Kelch 13* (*PfK13*) gene was assessed using nested PCR followed by amplicon sequencing.

## Specifications Table

**Table:** 

Subject	Parasitology
Specific subject area	Antimalarial drug resistance
Type of data	Table Image Chart Graph Figure
How data were acquired	DNA was extracted and Polymerase chain reaction followed by high resolution melt on a LightScanner 384 system (BioFire Diagnotics, inc., Salt Lake USA) and sequencing using ABI 3500XL Genetic Analyser (Applied Biosystems, Foster, USA) were used for analysis.
Data format	Raw Analyzed
Parameters for data collection	Genomic DNA was extracted from Dried blood spots were collected during the survey. The extracted DNA was analysed using Photo-induced electron transfer-PCR (PET-PCR) for malaria positivity and species identification. Only Plasmodium falciparum positive samples were analysed for resistance markers
Description of data collection	Data was collected through a household survey from Southern and Western Provinces in Zambia. Sample collected from the survey were used for this analysis.
Data source location	Institution: Ministry of Health-National Malaria Elimination Centre City/Province: Lusaka/Lusaka Country: Zambia Primary data sources:] Ministry of Health Mass drug administration survey of 2017 for Southern and Western Provinces
Data accessibility	Repository name: mendeley Data identification number: DOI:10.17632/zfk9brr5d9.1 Direct URL to data: https://data.mendeley.com/datasets/zfk9brr5d9/1
Related research article	L. Sitali, M. C. Mwenda, J. M. Miller, D. J. Bridges, M. B. Hawela, B. Hamainza, M. Mudenda-Chilufya, E. Chizema-Kawesha, R. Daniels, T. P. Eisele, A. H. Nerland, J. Chipeta, B. Lindtjørn (2020) Surveillance of antimalarial drugs in Zambia: Surveillance of Molecular Markers for Antimalarial Resistance in Zambia: Polymorphism of Pfkelch 13, Pfmdr1 and Pfdhfr/Pfdhps genes, Acta Tropica. 2020 Sept. 105704. DOI https://doi.org/10.1016/j.actatropica.2020.105704

## Value of the Data

• This data is important for the monitoring of antimalarial drug resistance.
• This data can guide policy makers on the resistance pattern of the currently used antimalarial.
• The data can be used for further studies on resistance makers especially in systematic review and meta-analysis.
• The data add to the body of information of mutations in the *Pfdhfr* and *Pfdhps* genes of SP, Pf *mdr-1* related to lumefantrine sensitivity and *Pfkelch 13* related to artemether resistance.

## 1 Data Description

The data set (https://data.mendeley.com/datasets/zfk9brr5d9/1) consist of results obtained from HRM-PCR technic from samples collected from the Western and Southern Provinces. The results were from an analysis of three gene *Pfdhfr* (51, 59, 108 and 164), *Pfdhps* (436, 437, 540 and 581) and *Pfmdr* (86,184 and 1246). The wild type is indicated as ‘1’, while the mutant ‘0’. The data shows wild type, mutant and mixed infections.

The abbreviations in the dataset are as follows: W-wild type; M-Mutant; I-Isoleucine; L-Leucine; S-Serine; N-Asparagine; C-Cysteine; R-Arginine; E-Glutamic acid; K-Lysine; A-Alanine; G-Glycine; Y-Tyrosine; D=Aspartic acid; F-Phenylalanine; dhfr-dihydrofolate reductase; dhpsdihydropteroate synthase; M-Male; F-Female; Haplo-Haplotype

**Fig. 1:**
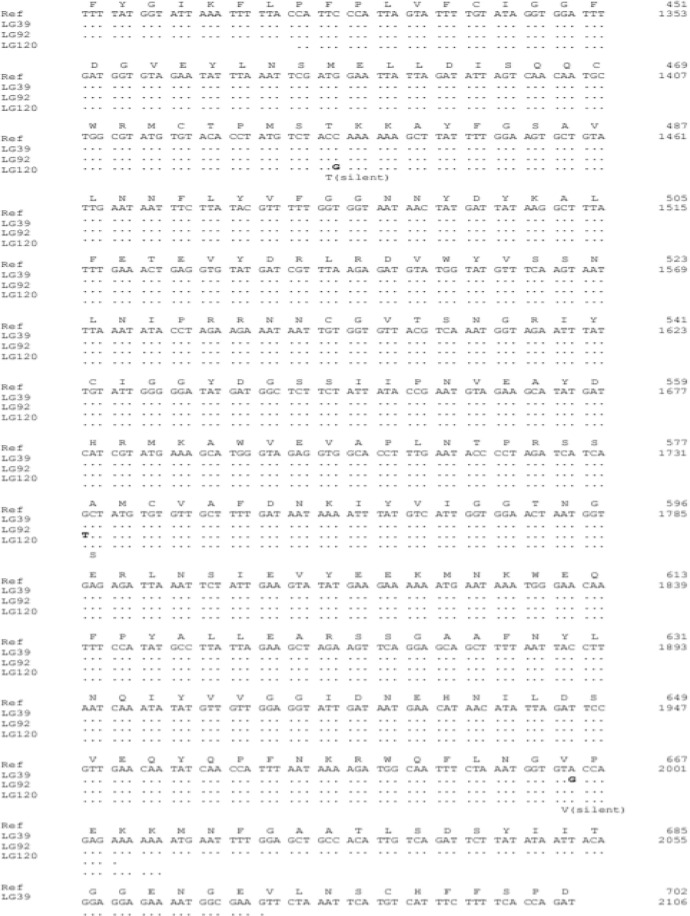
Nucleotide sequence for *PfKelch 13* and deduced amino acid sequences, showing the three samples that had mutations.

The table in the article (doi:10.1016/j.actatropica.2020.105704) related to this data in brief [1] as supplementary information, shows the frequencies of the *Pfdhfr, Pfdhps* and *Pfmdr1* single nucleotide polymorphisms for the samples from Southern and Western Provinces. The most prevalent mutant alleles observed were: *Pfdhps* 437G (87.7%), followed by *Pfdhfr* 59R (81.3%), 51I (66.7%) and 108N (66.8%). The other observed mutant alleles or point mutations were at low frequencies. Mixed alleles were also observed in all the genes with exception isolates from Southern, where *Pfdhps* 436 and 581 did not have mixed alleles. It is difficult to compare the two provinces as the sample sizes were very different, Southern has a small number of positives samples. For *Pfmdr*, no mutations were obsrved in Pfmdr 1 N86Y while Y184F has 33.3% mutations. In the case of *Pfkelch 13*, out of the 80 sequenced samples, only 3 has mutations (Fig. 1). In Figure the frequency of mutations is shown 4.1%.

**Fig. 2:**
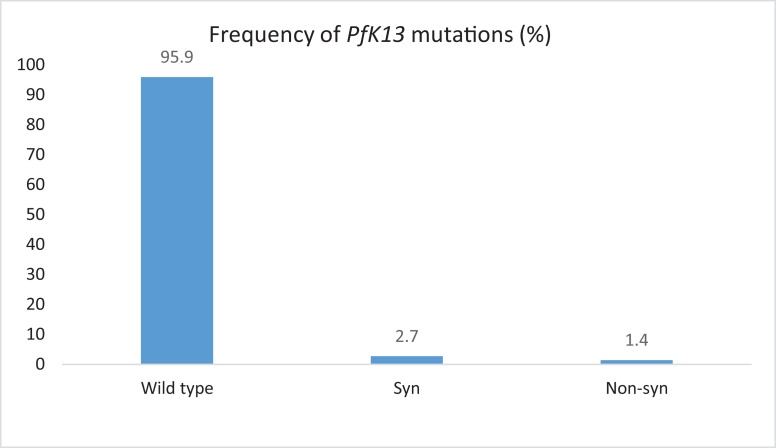
*Pfkelch 13* mutations.

## 2 Experimental Design, Materials and Methods

### 2.1 Polymerase chain reaction (PCR)

#### 2.1.1 DNA extraction

Genomic DNA was extracted from 6 mm DBS punches, approximately 13.8 µl whole blood, using a Qiagen DNA mini kit (Qiagen, Hilder, Germany) and eluted in 100 µl elution buffer. The puncher was cleaned after every sample by dipping in 70% ethanal and flaming. The extraction process for RDT positive and negative samples was different. RDT-positive samples were extracted individually, while RDT-negatives were extracted in pools of 10, and pools that came out positives were deconvoluted and re-extracted individually. The extracted DNA was stored at 4 °C for immediate analysis.

#### 2.1.2 PET-PCR analysis

Samples were run using PET-PCR and PCR-HRM. PET-PCR was performed on a LightCycler LC 480 real-time PCR machine (Roche, Basel, Switzerland), as described in 2013 by Lucchi *et al* [2]. To amplify *Plasmodium*18S ribosomal RNA the primer shown in Table 2 were used. Briefly, 5µl of DNA template was amplified in a 20 µl reaction volume containing 1x of the Taqman 2X environmental master mix (Applied Biosystems, Life Technologies LTD, Warrington, UK) as follows: 95 °C for 15 min, followed by 45 cycles of 95 °C for 20 s, 60 °C for 40 s and 72 °C for 30 s. Samples were tested in duplicate and recorded positive if both duplicate samples had a cycle threshold (CT) value < 40.

**Table 2:** Primer sequences for species identification.

Primer name	Sequence (5’ – 3’)
Original Genus 18sFor	GGC CTA ACA TGG CTA TGA CG
Original Genus FAM 18sRev	FAM-aggcgcatagcgcctggCTGCCTTCCT TAG ATGTGG TAG CT
Falciparum For	ACC CCTCGCCTG GTG TTT TT
Falciparum Rev	HEX-aggcggataccgcctggTCGG GCC CCA AAA ATA GGA A
*P. vivax* For	GTA GCC TAAGAAGGC CGT GT
*P. vivax* Rev	HEX-aggcgcatagcgcctggCCTGGGG GAT GAA TAT CTC TAC AGC ACT GT
*P. malariae* For	AAGGCAGTAACACCAGCAGTA
*P. malariae* Rev -based on dihydofolate reductase-thymidylate synthase (DHFR-TS) gene	FAM-aggcgcatagcgcctggTCCCATGAAGTTATATTCCCGCTC
*P. ovale* For	FAM-aggcgcatagcgcctggCCACAGATAAGAAGTCTCAAGTACGATATT
*P. ovale* Rev	TTGGAGCACTTTTGTTTGCAA

*Note: the lowercase letters represent sequences with non-homology to the template DNA

#### 2.1.3 HRM analysis for Pfdhfr, Pfdhps and Pfmdr 1

*Pre-amplification:* The PCR-HRM analysis started with a pre-amplification process to enhance the template concentrations. The pre-amplification was performed on all *P. falciparum* positive samples. A pre-amplification master (PreAmp Master Mix, Life Technologies, Inc, Grand Island, NY, USA) was used with a mixture of primers for the assays that were run. DNA from samples with a CT value of > 35 was pre-amplified in a 10 µl reaction volume and the ones with CT < 35, in a 20 µl reaction volume. The following were pre-amplification conditions; pre-incubation 95 °C for 10 min, followed by 14 cycles of amplification for 15 min and annealing for 4 min; and final extension for 15 min. After pre-amplification, the DNA was cleaned using a Zymo kit-ZR-96 DNA sequence Clean-up Kit (Catalogue No. D 4053, Zymo research, Tustin, CA, USA).

*PCR amplification and HRM:* All PCR amplifications were performed on the LightCycler 480 real-time PCR machine. The reaction consisted of 2.0 µL of Lightscanner Master Mix, 2.5 µL of the pre-amplified template, 0.5 µL of primers and probes (Final primer/probe concentrations for a 5-µL total reaction volume was 0.5 µM excess primer, 0.1 µM limiting primer, and 0.4 µM of the 3′-blocked probe). The list of primers and probes are shown in Table 3. Specific controls for wild type or mutant genes were included for each assay. The amplification conditions were as follows: 95 °C denaturation for 2 min, 50 cycles of 94 °C for 5 s and 66 °C for 30 s, and a pre-melt cycle of 5 s each at 95 °C and 37 °C. The product was heated from 40 °C to 90 °C on the Lightscanner system and the change in fluorescence was recorded as the samples melted incrementally. The following assays were run Pfdhfr (N51I, C59R, I164L and S108N); Pfdhps (S436F, A437G, K540E/N and A581G); and *Pfmdr* (N86F, Y184F and D1246Y. The annealing temperature for all assays was 66 °C with the exception of two assays S108N and D1246Y that were run at 63 °C [3,4].

**Table 3:** High resolution melting assays primer and probe sequences.

	Forward Primer 5′→3′	Reverse Primer 5′→3′	Probe 5′→3′
Pfcrt N75/K76	GTAAAACGACGGCCAGTTTCTTGTCTTGGTAAATGTGCTCA	CAGGAAACAGCTATGACCGGATGTTACAAAACTATAGTTACCAAT	GTGTATGTGTAATGAATAAAATTTTTG-block
Pfdhfr N51/C59	ACATTTAGAGGTCTAGGAAATAAAGGAGT	ATATTTACATCTCTTATATTTCAATTTTTCATATTTTGATTCATTCAC	AAATGTAATTCCCTAGATATGAAATATTTTTGTGCAG-block
Pfdhfr I164	ACAAAGTTGAAGATCTAATAGTTTTACTTGGG	CTGGAAAAAATACATCACATTCATATGTACTATTTATTCTA	AATGTTTTATTATAGGAGGTTCCG-block
Pfdhfr S108	CTGTGGATAATGTAAATGATATGCCTAATTCTA	GACAATATAACATTTATCCTATTGCTTAAAGGT	GGAAGAACAAGCTGGGAAAGCAT-block
Pfdhps S436/A437	GAATGTTTGAAATGATAAATGAAGGTGCTA	CAGGAAACAGCTATGACGAAATAATTGTAATACAGGTACTACTAAATCTCT	ATCCTCTGGTCCTTTTGTTATACC-block
Pfdhps K540	GTGTTGATAATGATTTAGTTGATATATTAAATGATATTAGTGC	GTTTATCCATTGTATGTGGATTTCCTCTT	TAATCCAGAAATTATAAAATTATTAAAAAAAAAAAAC-block
Pfdhps A581	CTTGTATTAAATGGAATACCTCGTTATAGGA	AGTGGATACTCATCATATACATGTATATTTTGTAAG	TTGGATTAGGATTTGCGAAGAAACATGATCA-block
Pfmdr N86	TTATTATTTATATCATTTGTATGTGCTGTATTATCAGG	CAGGAAACAGCTATGACATCATTGATAATATAAATTGTACTAAACCTATAGATACT	GAACATGAATTTAGGTGATGATATTAATCC-block
Pfmdr Y184	AGTTCAGGAATTGGTACGAAATTTATAACA	ACGCAAGTAATACATAAAGTCAAACG	CCTTTTTAGGTTTATATATTTGGTCAT-block
Pfmdr D1246	GCAGAAGATTATACTGTATTTAATAATAATGGAGA	TTTCATATATGGACATATTAAATAACATGGGT	GTGATTATAACTTAAGAGATCTTAGAAACT-block

#### 2.1.4 Kelch-13 propeller domain amplification and sequencing

Amplification of the Kelch 13 Propeller gene was performed using nested PCR and primers in Table 4 were used. For both primary and nested reactions, the total reaction volumes were 25 µL and 50 µL respectively. The reaction contained 1x final concentration buffer, 2.5 mM MgCl_2_, 20 nM of each dNTPs and 1.25 U Taq® polymerase (Solis BioDyne, Estonia), 250 nM of each primer, with 2.5 template (25 µL reaction volume) and 5 µL template (50 µL reaction volume). For both the primary and nested reactions, 30 cycles were undertaken on a MyCycler™ thermal cycler (Bio-Rad Laboratories, USA) as follows: 15 min at 95 °C, then 30 cycles of 30 s at 95 °C, 2 min at 58 °C, 2 min at 72 °C and final extension 10 min at 72 °C, for the primary reaction. For the nested reaction the PCR conditions were 15 min at 95 °C, then 40 cycles of 30 s at 95 °C,1 min at 60 °C, 1 min at 72 °C and final extension 10 min at 72 °C.

**Table 4:** Primer sequences for K13 propeller gene.

Procedure (size)	Primer sequence	Size
First PCR	K13 F1 5′-CGGAGTGACCAAATCTGGA-3′ K13 R1 5′-GGGAATCTGGTGGTAACAGC-3′	2097 bp
Nested PCR	K13 F2 5′-GCCAAGCTGCCATTCATTTG-3′ K13 R2 5′-GCCTTGAAAGAAGCAGA-3′	850 bp
Sequencing	K13 F3 5′-TTATGTCATTGGTGGAACTAA-3′ K13 R3 5′- TCTAGGGGTATTCAAAGGTGC-3′	

Sequencing of *Pfk13* was performed in South Africa at Inqaba Biotechnology industries. The labelled products were cleaned using the ZR-96 DNA sequence Clean-up Kit (Catalogue No. D4053, Zymo research). The cleaned products were then analysed using the Applied Biosystems ABI 3500XL Genetic Analyser (Themofisher).

## Data Analysis

Allele prevalence was analysed using Stata version 13 (College Station, TX, USA). Any sample that contained a mixed result (i.e. presence of both wild-type and mutant alleles) was scored as a mutant. The graph was prepared in excel. Multiple nucleotide sequence alignments were analysed by MacVector (Cambridge, UK) using the 3D7 *PfK13* sequence (GenBank accession no. XM001350122) as a reference to detect point mutations in the gene.

## Ethics Statement

Ethical clearance was sought from the Regional Committee for Medical and Health Research Ethics- Western Norway Ref no. 2016/1393/REK Vest (Norway) and from the University of Zambia Biomedical Research Ethics Committee (UNZABREC) (Zambia) Ref no. 010-05-16. This study was an analysis of samples from a larger study, that was assessing progress made in malaria control in Zambia, thus ethical clearance for the larger study was earlier obtained from UNZABREC, ref no. 007-03-14. Permission to use the Ministry of Health data was obtained from the National Health Research Authority. All data analysed were anonymized. Consent was obtained during the data collection from the participants involved in the study.

## Credit author statement

**Lungowe Sitali:** conceptualization, data curation, formal analysis, investigation, methodology, project administration, visualisation, writing-original draft, writing-review and editing. **Mulenga C. Mwenda**: formal analysis, investigation, methodology, project administration, validation, visualisation, writing-original draft, writing-review. **John M. Miller:** data curation, formal analysis, fund acquisition, investigation, methodology, project administration, resources, writing-original draft, writing-review and editing. **Daniel J. Bridges:** data curation, investigation, methodology, project administration, software, writing-original draft, writing-review and editing. **Moonga B. Hawela:** formal analysis, investigation, methodology, writing-original draft, writing-review. **Busiku Hamainza:** formal analysis, investigation, methodology, writing-original draft, writing-review**. Mutinta Mudenda-Chilufya:** formal analysis, investigation, methodology, project administration, resources, writing-original draft, writing-review. **Elizabeth Chizema-Kawesha:** formal analysis, investigation, methodology, project administration, resources, writing-original draft, writing-review. **Rachel F. Daniels:** investigation, methodology, software, project administration, visualisation, writing-original draft, writing-review and editing **Thomas P. Eisele:** data curation, investigation, methodology, resources, writing-original draft, writing-review and editing. **Audun H. Nerland:** investigation, methodology, project administration, software, visualisation, writing-original draft, writing-review and editing **James Chipeta:** conceptualization, formal analysis, investigation, methodology, project administration, visualisation, supervision, writing-original draft. **Bernt Lindtjorn:** conceptualization, data curation, fund acquisition, investigation, methodology, project administration, visualisation, supervision, writing-original draft, writing-review and editing.

## Declaration of Competing Interest

The authors declare that they have no known competing financial interests or personal relationships which have, or could be perceived to have, influenced the work reported in this article.

## References

[1] Sitali L., Mwenda M.C., Miller J.M., Bridges D.J., Hawela M.B., Hamainza B., Mudenda-Chilufya M., Chizema-Kawesha E., Daniels R.F., Eisele T.P. (2020). Surveillance of molecular markers for antimalarial resistance in Zambia: polymorphism of Pfkelch 13, Pfmdr1 and Pfdhfr/Pfdhps genes. Acta Tropica.

[2] Lucchi N.W., Narayanan J., Karell M.A., Xayavong M., Kariuki S., DaSilva A.J., Hill V., Udhayakumar V. (2013). Molecular diagnosis of malaria by photo-induced electron transfer fluorogenic primers: PET-PCR. PLoS One.

[3] Obaldia N., Baro N.K., Calzada J.E., Santamaria A.M., Daniels R., Wong W., Chang H.H., Hamilton E.J., Arevalo-Herrera M., Herrera S., Wirth D.F., Hartl D.L., Marti M., Volkman S.K. (2015). Clonal outbreak of Plasmodium falciparum infection in eastern Panama. J. Infect. Dis..

[4] Daniels R., Ndiaye D., Wall M., McKinney J., Sene P.D., Sabeti P.C., Volkman S.K., Mboup S., Wirth D.F. (2012). Rapid, field-deployable method for genotyping and discovery of single-nucleotide polymorphisms associated with drug resistance in Plasmodium falciparum. Antimicrobial Agents Chemo..

